# miR-1247-3p targets STAT5A to inhibit lung adenocarcinoma cell migration and chemotherapy resistance

**DOI:** 10.7150/jca.65167

**Published:** 2022-03-28

**Authors:** Jiansheng Lin, Xinyang Zheng, Xikun Tian, Jun Guan, Haizhan Shi

**Affiliations:** 1 Department of cardiothoracic surgery, The First Hospital of Putian City, No 449 Nanmen West Road, 351100, Chengxiang District, Putian City, Fujian Province, China.; 2 Chosenmed technology (Beijing) Co., Ltd, No. 156, Jinghai 4th Road, Tongzhou District, Beijing, China.

**Keywords:** miR-1247-3p, lung adenocarcinoma, STAT5A, cell migration, chemoresistance

## Abstract

**Background:** Although several advancements have been achieved in research and treatment of lung adenocarcinoma in the past few years, the mechanism concerning cancerous cell migration and the cause of chemoresistance remains ambiguous. This research aimed to explore the impact of miR-1247-3p in lung adenocarcinoma.

**Methods:** The mRNA expression of miR-1247-3p and STAT5A were conducted with qRT-PCR. Lentiviral vectors containing miR-1247-3p mimics and inhibitors were constructed. Cell migration were examined using Transwell assay. To observe chemotherapy resistance, Docetaxel, Doxorubicin, and Gefitinib were used. DIANA, miRDB, and TargetScan databases were applied to detect target genes. The binding sites were verified by double luciferase assay.

**Results:** Low expression of miR-1247-3p was observed in lung adenocarcinoma tissues and cell lines. Its expression was lower in advanced stages. Cell migration of lung adenocarcinoma was inhibited by miR-1247-3p, and it could negatively regulate the process of chemoresistance. miR-1247-3p directly binds to 3' UTR of STAT5A mRNA, and it functions via targeting STAT5A.

**Conclusions:** miR-1247-3p acted as a potential governor monitoring cell migration and chemotherapy resistance of LUAD by interacting with STAT5A. It has the potential to be exploited as novel therapeutic target for LUAD in the future.

## Introduction

Currently, the highest incidence and mortality of cancer is lung cancer 1. Lung cancer is lower than 20 percent in 5-year survival rate 2. The most common histological subtype is lung adenocarcinoma (LUAD), accounting for around 40% of lung malignancies 3. The most common risk factor of LUAD is smoking. Long-term exposure to external air pollution, carcinogens, and radon are other risk factors. Diagnoses of cancer in non-smokers have risen significantly in recent years, stressing the importance of risk factors for non-smoking in the genesis of LUAD 4. EGFR, STK11, KEAP1, KRAS, and TP53 are common somatic mutations in LUAD. Female non-smokers having EGFR mutation represent a significant percentage of LUAD cases 5. Immunotherapy, chemotherapy, radiation, surgery, or a combination of these therapies, are the therapeutic choices for LUAD 2, 6. A recent development in targeted therapies has achieved significant success targeting many oncogenic drivers. For the precise treatment of LUAD, immune checkpoint inhibitor that target PD-1 or PD-L1^7^, ALK and ROS1 8, and inhibitors against BRAF V600E and EGFR mutations 9 were developed. There have also been potentially good results shown in other therapies pointing at ERBB2 (HER2) mutations, RET rearrangement, and MET amplification 10. In the tumor microenvironment, the impact of immunotherapy on LUAD development and consequence depends on the tumor-infiltrating immune cell subsets and the cancer phenotype 11. In spite of these advancements, due to the complexity of targeting oncogenic mutations or the lack of identified genetic mutations, a significant percentage of LUAD with no targeted therapeutic options available is still present. In addition, acquired and intrinsic resistance to targeted therapies is normally seen in patients with lung adenocarcinoma 12.

MicroRNAs are small, non-coding RNAs, 20 to 24 bp in length, and act by interacting with the target mRNAs. It binds to the 3'-UTR and negatively regulates the target gene transcription 13-15. It was shown in previous researches that in many cancers, miR-1247-3p was abnormally expressed and had various functions 16. For instance, in breast cancer, miR-1247 was increased in cancerous tissues, in contrast with similar adjacent normal tissues 17. In pancreatic cancer, miR-1247-3p inhibits cell proliferation by attacking neuropils 18. Moreover, in osteosarcoma, it is downregulated and appears to prohibit tumor growth 19. Recently, miR-1247 has been found to initiate activation of fibroblasts associated with cancer in liver cancer metastases. miR-1247-3p could lead to the activation of β1-integrin-NF-κB signaling in fibroblasts through B4GALT3 20. The involvement of miR-1247 in bladder cancer has also been observed 21.

Ultimately, a complete understanding of lung adenocarcinoma's molecular mechanisms is extremely important. In the current article, we studied the underived mechanism of miR-1247-3p in LUAD, the results might empower development of new therapeutic methods in the coming future.

## Material and Methods

### Clinical sample

This study conformed to the declarations of Helsinki. The institutional research ethics committee of The First Hospital of Putian approved this study. 162 patients diagnosed with lung adenocarcinoma were selected (Table 1). Every patient participated with their own consent and went through surgery. From each patient, both cancerous and normal tissue samples were collected. These tissues were then frozen, then immediately kept in the fridge. The inclusion criteria of enrolled patients: the lung adenocarcinoma patients confirmed via biopsy and histological detection. The exclusion criteria: (1) Severe mental disorders; (2) clinically immunodeficiency disease; (3) pregnant woman.

### Cell culture

We purchased cancerous cell lines of human adenocarcinoma (NCI-H1299, NCI-H1395, A549, NCL-H460, PG49, NCI-H1993) and a normal cell line (HNBE) from Shanghai Huiying Bio-technology Company. These cells were grown in RPMI 1640 medium (#R8758, Sigma, USA) having 10% fetal serum (100 Ug/ml penicillin and 100 ug/ml streptomycin). They were positioned in an incubator at 37 °C temperature with 5% CO_2_ humid environment for culturing.

### Bioinformatics methods

We used miRDB database 22, DIANA database 23, and TargetScan 24 database for predicting target genes of miR-1247-3p.

### qRT-PCR

Through using Trizol reagent (#10296010, Thermo Fisher, USA), we extracted total RNA from tissues and cell lines. Reverse transcription was carried out using TaqMan microRNA Reverse Transcriptase Kit (#4366596, Thermo Fisher, USA). We performed PCR on the Prism 7500 Fast sequence detection system (Applied Biosystems).

### Western blot

Cell protein was confined with 1% PMSF and RIPA lysine buffer (#89900, Thermo Fisher, USA) and further reacting with SDS-PAGE buffer. Proteins were then relocated to the poly-difluoroethylene layer. After culturing at room temperature for 1 hour, it grew at night. An ECL chemiluminescent kit (#32209, Thermo Fisher, USA) in which the protein and the secondary antibody were fried for 1 hour was used. Then the Gene Gnome 5 test was applied for examining the bands.

### Transwell assay

We used Transwell assay for examining the ability of cell migration and invasion. Upper and lower chambers were separated by placing 8-μm pore size inserts. 1×10^5^ cells were added into the upper chambers with non-coated membranes, and a cell culture medium having 20% FBS (Fetal Bovine Serum) was added in the bottom chambers. After incubating at 37 °C temperature and 5% CO_2_ atmosphere for 1 day, the cells that emigrated into the lower chamber were fixed in 4% methanol for 20 minutes. Then they were stained with 0.1% crystal at room temperature for 10 minutes. Subsequently, we used an Olympus microscope to count the cells from five randomly selected fields.

### Lentiviruses and stable transfection

miR-1247-3p mimic and inhibitor with their parallel negative controls, mNC, and inhibitor NC respectively, were purchased. Vectors were transfected, and plasmids were packaged into cells using Polyget Regent. Virus supernatants were collected after 48 hours. Cancerous cells were spin-infected in 12-well plated. Then we performed centrifugation of lentiviral supernatants for 30 minutes. Cells were selected using antibiotic puromycin (3 ng/mL) to harvest stable cell lines. Lipofectamine 2000 (#11668019, Thermo Fisher, USA) was used to conduct transient transfections.

### Dual-luciferase reporter assay

Two days after transfection, signals were identified using a dual-luciferase reporter assay kit following the protocols provided by the manufacturer. We performed three independent trials and the data was shown as mean± SD.

### Immunohistochemistry staining

Tissues were fixed using 4% formalin for 48 h. Then, the tissues were embedded using OCT (Sigma, USA). The tissues were sectioned in 8 μm thickness. After antigen repair, 3% H_2_O_2_ treatment, washing with PBS, and blocking with 5% goat serum. The primary antibodies (Rabbit monoclonal to c-myc, 1:1500, #ab32072, Abcam; Rabbit monoclonal to axin, 1:1000, #ab109307, Abcam; Rabbit monoclonal to beta catenin, 1:1000, #ab32572, Abcam) were used to cultivate tissues at 4 °C overnight. After washing, the tissues were cultured with second antibody (Goat anti-rabbit lgG, 1:2000, #ab205718, Abcam) for 2 h. DAB reagent (#34002, Thermo Fisher, USA) was used to treat tissues. The sections were visualized and captured using an inverted optical microscope (Olympus Corporation). The expression intensity was analyzed through Image J software.

### Transplanted tumor model

6×10^6^ NCI-H446 cells (0.1 mL) were subcutaneously injected into the armpits of mice (Vital River Laboratory Animal Technology, Beijing, China). The animals were divided into 2 groups randomly When the tumor grew to 6 mm. Animals in the group were STAT5A siRNA subcutaneously treated with STAT5A siRNA (5 µM, 0.2 mL), and the mice in the group control were treated with same amount of normal saline once a day. After 21 days, mice were sacrificed, and the tumor weight was measured.

### Statistical analysis

SPSS 25.0 was used for statistical analysis of data and variations between two groups were compared using t test. One-way ANOVA was used to analyze the data of multiple groups, followed by Dunnett's t-test. To calculate correlation between miR-1247-3p and STAT5A, Spearman's rank test was used.

## Results

### Low expression of miR-1247-3p was found in adenocarcinoma tissues of lung

**Figure 1:**
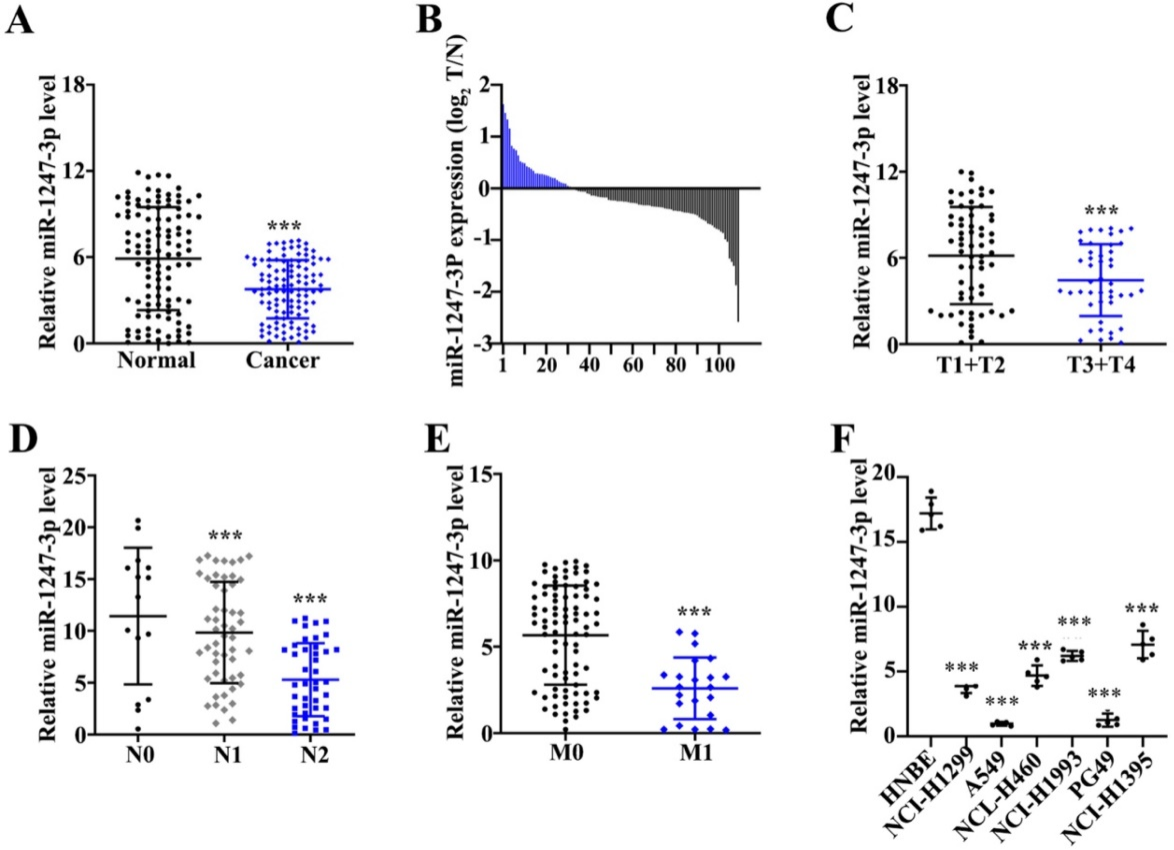
** Low expression of miR-1247-3p was found in lung adenocarcinoma tissues.** A:162 pairs from adenocarcinoma tissues of lung were examined and miR-1247-3p expression was observed using qRT-PCR. Unlike adjacent normal tissue cells, a reduced miR-1247-3p expression was found in these tissues. B: Among these 162 pairs, 113 tumor tissues showed low and 7 showed high expressions of miR-1247-3p. C: miR-1247-3p expression in T1+T2 vs T3+T4 in adenocarcinoma tissues of lung was observed. Results revealed a higher expression in T1+T2 as compared to T3+T4. D: The expression of miR-1247-3p was also detected in adenocarcinoma tissue of lung with or without lymph nodes tumors. E: The expression of miR-1247-3p was also detected in adenocarcinoma tissue of lung showing distant metastasis and also in the tissues without showing any symptom of distant metastasis. F: miR-1247-3p expression was also detected in normal epithelial cells of lung and cell lines of adenocarcinoma tissue cells. The expression was higher in normal epithelial cells of lung. ***p<0.001 compared with group normal, T1+T2, N0, M0, or HNBE. Lentiviruses-inhibitor normal control (LV-INC), Lentiviruses-inhibitor (LV-inhibitor), Lentiviruses-mimics normal control (LV-mNC), Lentiviruses- mimics (LV-mimics). The experiments were repeated at least 3 independent times.

Unlike adjacent normal tissue cells, a reduced miR-1247-3p expression was found in tumor tissues (p<0.001) (Figure 1A). Among these 162 pairs, 113 tumor tissues showed low and 7 showed high expressions of miR-1247-3p (Figure 1B). miR-1247-3p expression in T1+T2 vs T3+T4 of lung adenocarcinoma tissues was observed (Figure 1C). Results revealed a higher expression in T1+T2 as compared to T3+T4 (p<0.001). miR-1247-3p expression was also detected in lung adenocarcinoma with or without lymph nodes metastasis and it was higher without lymph node metastasis (p<0.001) (Figure 1D). Significant higher expression of miR-1247-3p in lung adenocarcinoma without distant metastasis was observed (p<0.001) (Figure 1E). miR-1247-3p expression was also detected in normal epithelial cells of lung and four cell lines of adenocarcinoma tissue cells. The expression of miR-1247-3p was significant higher in HNBE cell lines (p<0.01) (Figure 1F).

### Lung adenocarcinoma cell proliferation was inhibited by miR-1247-3p

**Figure 2:**
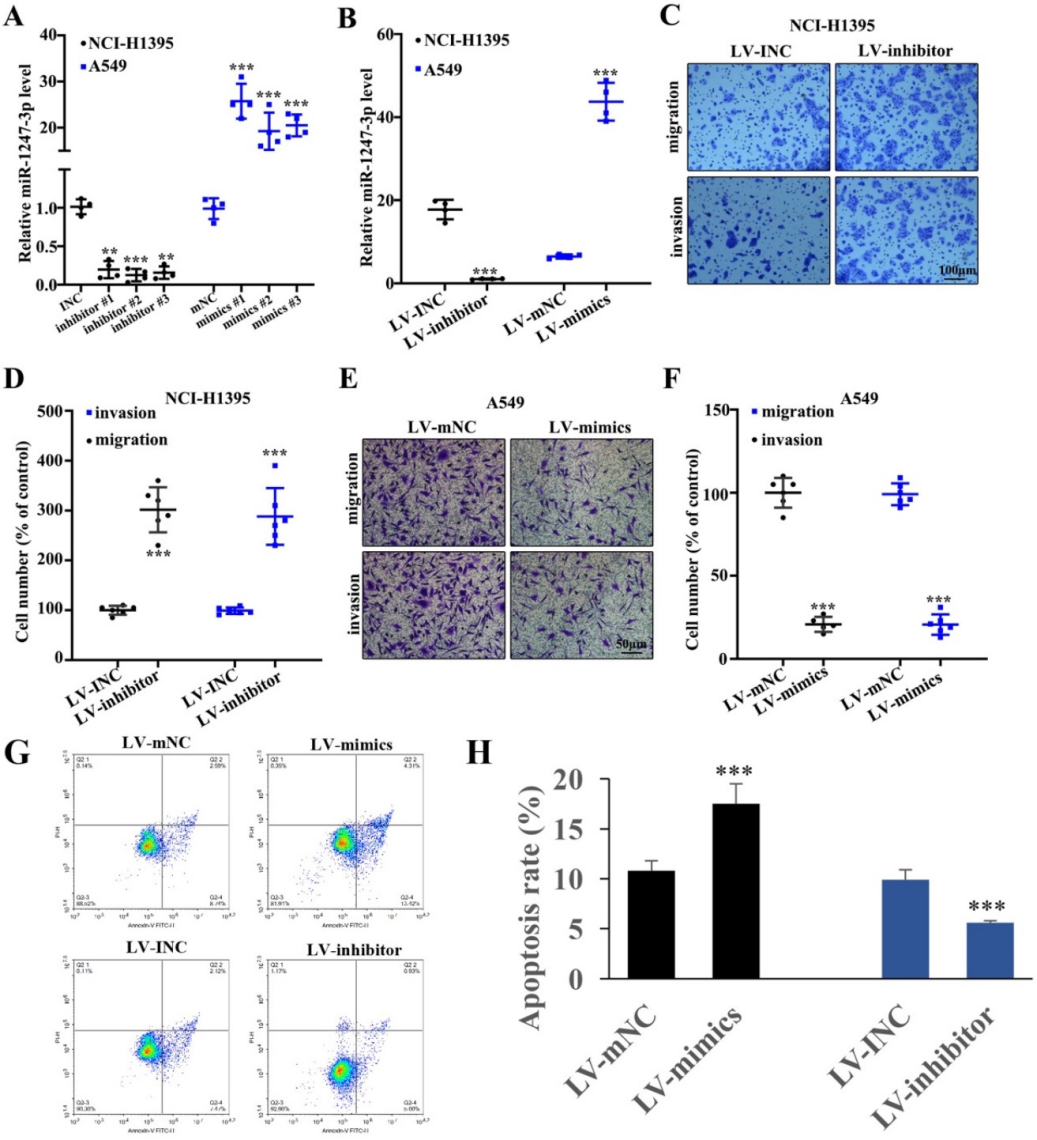
** Adenocarcinoma cell proliferation of lung was inhibited by miR-1247-3p. A:** We identified the best mimic and inhibitor sequences. **B:** As compared to control groups, a lower expression of miR-1247-3p was observed in inhibitor group and higher in mimic group. **C:** Reduced expression of miR-1247-3p resulted in an increased cell proliferation and invasion. **D:** Suppression process can result in increment of cell number as shown. LV-mimics group was found to be decreased in cell number of A459 cell lines whereas it was higher in LV-mNC group. **E-F:** Cell number percent in A549 cell lines was raised by inhibitor group when comparing to LV-INC group. **G-H:** Cell apoptosis was measured. ***p<0.001 compared with group INC, mNC, LV-INC, or LV-mNC. Lentiviruses-inhibitor normal control (LV-INC), Lentiviruses-inhibitor (LV-inhibitor), Lentiviruses-mimics normal control (LV-mNC), Lentiviruses- mimics (LV-mimics). The experiments were repeated at least 3 independent times.

To further explore the performance of miR-1247-3p in adenocarcinoma cells of lung, we suppressed its activity in NCI-H1395 cells and boosted it in A549 cells and this modification resulted in successful findings (p<0.001). Then we identified and separated the best mimic and inhibitor sequences (Figure 2A). In order to validate the efficacy of lentivirus transfection, polymerase chain reaction was performed. As compared to control groups, a lower expression of miR-1247-3p was observed in inhibitor group and higher in the control group (p<0.001) (Figure 2B). Knockdown treated NCI-H1395 cells were studied through Transwell assay. A reduced expression of miR-1247-3p resulted in an increased cell proliferation and invasion (Figure 2C-D). The mimics group was found to be lower in cell number percent in A459 cell lines whereas higher in LV-mNC group (p<0.001) (Figure 2E). The cell number percent in A549 cell lines was raised in inhibitors group as compared to LV-INC group (Figure 2F). Transfection with LV-mimics significantly promoted the cell apoptosis and LV-inhibitor suppressed cell apoptosis (Figure 2G-H).

### Chemotherapy resistance of adenocarcinoma cells of lung was inhibited by miR-1247-3p

**Figure 3:**
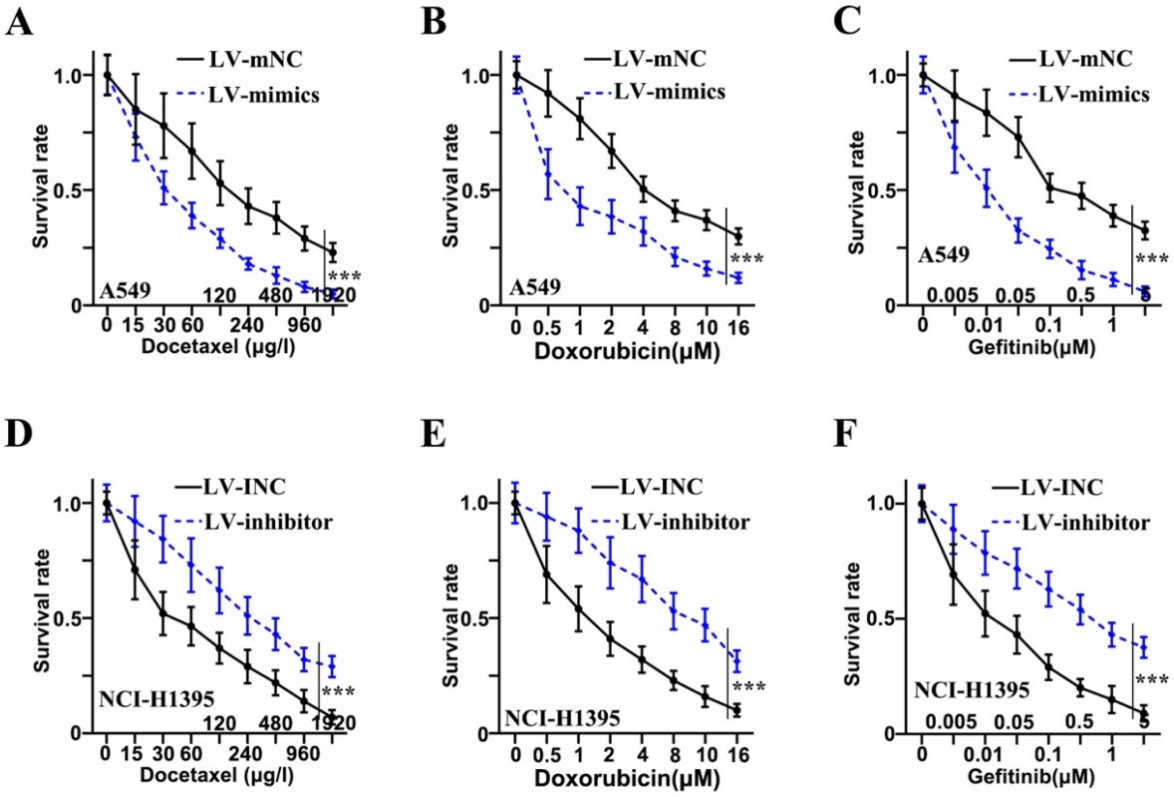
** Chemotherapy resistance of adenocarcinoma cells of lung was inhibited by miR-1247-3p. A:** When treated with Docetaxel, in the cell lines of A549, the mimics group was found to be in reduced survival rate as compared to the group of LV-mNC. **B:** When administered Doxorubicin, in the cell lines of A549, the mimics group was found to be in reduced survival rate when comparing with the LV-mNC group. **C:** When treated with Gefitinib, in A549 cells lines, LV-mimics group was lower in survival rate than LV-mNC group. **D:** In the cell lines of NCI-H1395, when treated with Docetaxel, a higher survival rate of inhibitor group was observed as compared to LV-INC group. **E:** In NCI-H1395 cell lines, when treated with Doxorubicin, LV-inhibitor group was increased in survival rate than LV-INC group. **F:** In NCI-H1395 cell lines, when treated with Gefitinib, a higher survival rate of inhibitor group was observed as compared to group of LV-INC. ***p<0.001 compared with group LV-INC or LV-mNC. Lentiviruses-inhibitor normal control (LV-INC), Lentiviruses-inhibitor (LV-inhibitor), Lentiviruses-mimics normal control (LV-mNC), Lentiviruses- mimics (LV-mimics). The experiments were repeated at least 3 independent times.

Three drugs (Docetaxel, Doxorubicin, and Gefitinib) were administered to examine the resistance to chemotherapy in adenocarcinoma cells of lung. Concerning Docetaxel, in the cell lines of A549, the mimics group was found to be in reduced survival rate as compared to the LV-mNC group and was shown in Figure 3A (p<0.001). In NCI-H1395 cell lines, a higher survival rate of inhibitor group was observed as compared to LV-INC group shown in Figure 3D (p<0.001). Concerning Doxorubicin, in the cell lines of A549, the mimics group was found to be in reduced survival rate as compared to the LV-mNC group and was shown in Figure 3B (p<0.001). In the cell lines of NCI-H1395, inhibitor group was higher in survival rate than LV-INC group (p<0.001) (Figure 3E). For Gefitinib, in A549 cells lines, mimics group was lower in survival rate than LV-mNC group (p<0.001) (Figure 3C), In the cell lines of NCI-H1395, a higher survival rate of inhibitor group was observed as compared to LV-INC group shown in Figure 3F (p<0.001).

### miR-1247-3p regulated STAT5A

**Figure 4:**
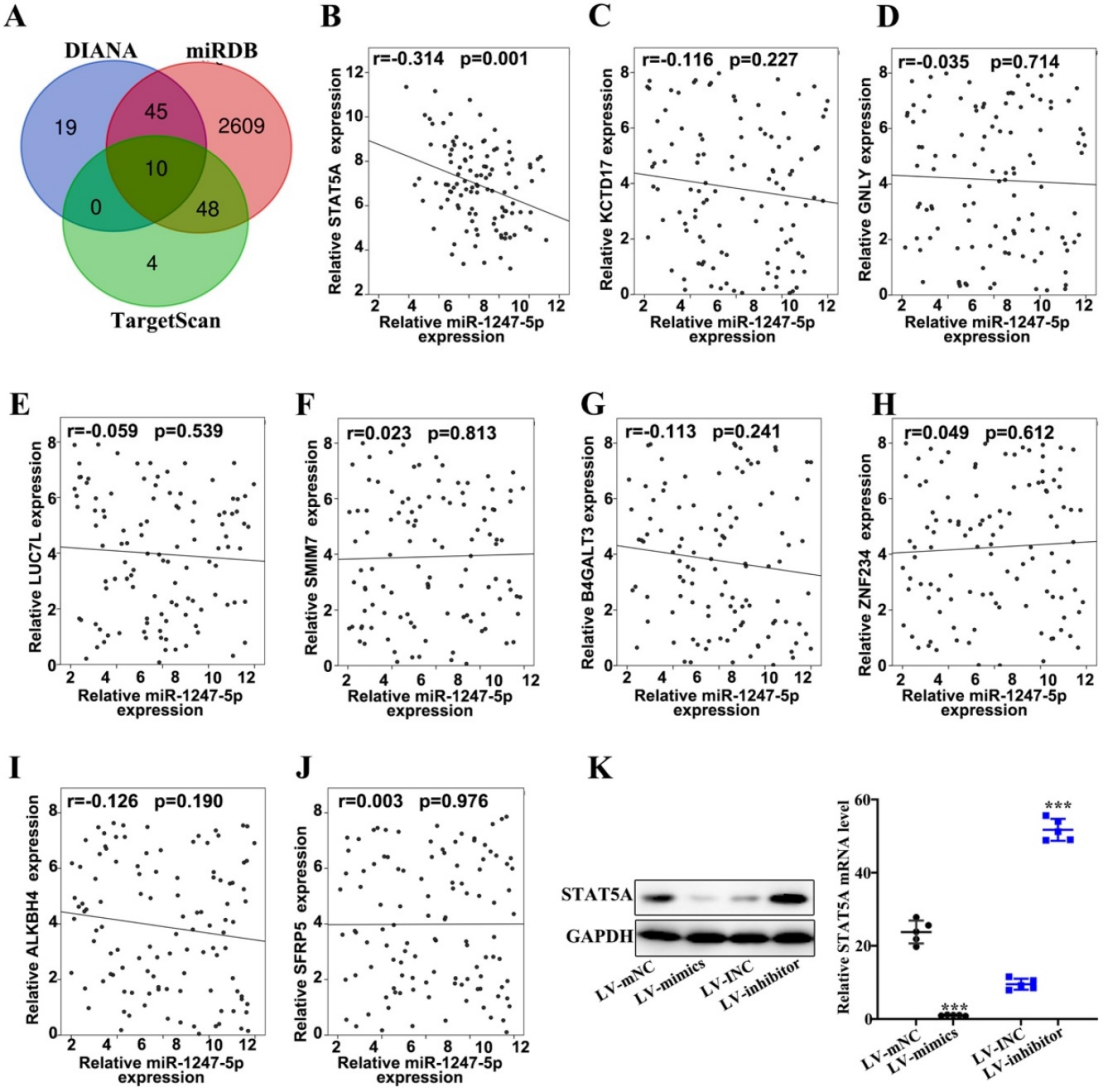
** miR-1247-3p regulated STAT5A. A.** Three databases (DIANA, miRDB, and TargetScan) were applied to prognosticate miR-1247-3p target genes. **B.** A negative correlation between relative microRNA-1247-5p expression and STATA5A expression (r=-0.314, p=0.001). **C.** Relative microRNA-1247-5p expression and KCTD 17 expression negatively correlated (r=-0.116, p=0.227). **D.** Relative microRNA-1247-5p expression and GNLY expression negatively correlated (r=-0.035, p=0.714). **E.** Relative microRNA-1247-5p expression and LUC7L expression negatively correlated (r=-0.059, p=0.539). **F.** A positive correlation between relative microRNA-1247-5p expression and SMIM7 expression (r=0.023, p=0.813). **G.** Relative microRNA-1247-5p expression and B4GALT3 expression negatively correlated (r=-0.113, p=0.241). **H.** A positive correlation between relative microRNA-1247-5p expression and ZNF234 expression (r=0.049, p=0.612). **I.** Relative microRNA-1247-5p expression and ALKBH4 expression negatively correlated (r=-0.126, p=0.190). **J.** A positive correlation between relative microRNA expression and SFRP5 expression (r=0.003, p=0.976). **K.** The expression of STAT5A was examined in sable transfected cell lines and was observed to be more in LV-mNC group than in mimics group (p<0.001). An enhanced expression of STAT5A was found in inhibitors group than the group of LV-INC. ***p<0.001 compared with group LV-INC or LV-mNC. Lentiviruses-inhibitor normal control (LV-INC), Lentiviruses-inhibitor (LV-inhibitor), Lentiviruses-mimics normal control (LV-mNC), Lentiviruses- mimics (LV-mimics). The experiments were repeated at least 3 independent times.

Three databases (DIANA, miRDB, and TargetScan) were applied to prognosticate target genes of miR-1247-3p (Figure 4A). Then we drew Venn diagram. Based on the final biological activity, STAT5A was selected and others were omitted. There was negative correlation between relative microRNA-1247-5p expression and STATA5A expression (r=-0.314, p=0.001) (Figure 4B). However, no significant correlation relationships were found between miR-1247-3p and KCTD 17 (Figure 4C), GNLY (Figure 4D), LUC7L (Figure 4E), SMIM7 (Figure 4F), B4GALT3 (Figure 4G), ZNF234 (Figure 4 H), ALKBH4 (Figure 4I), SFRP5 (Figure 4J). Meanwhile, the expression of STAT5A was examined in sable transfected cell lines and was observed to be elevated in LV-mNC group compared with the mimics group (p<0.001). An enhanced expression of STAT5A was found in the inhibitor group than LV-INC group (p<0.001) (Figure 4K).

### miR-1247-3p directly bound to STAT5A mRNA 3' UTR

**Figure 5:**
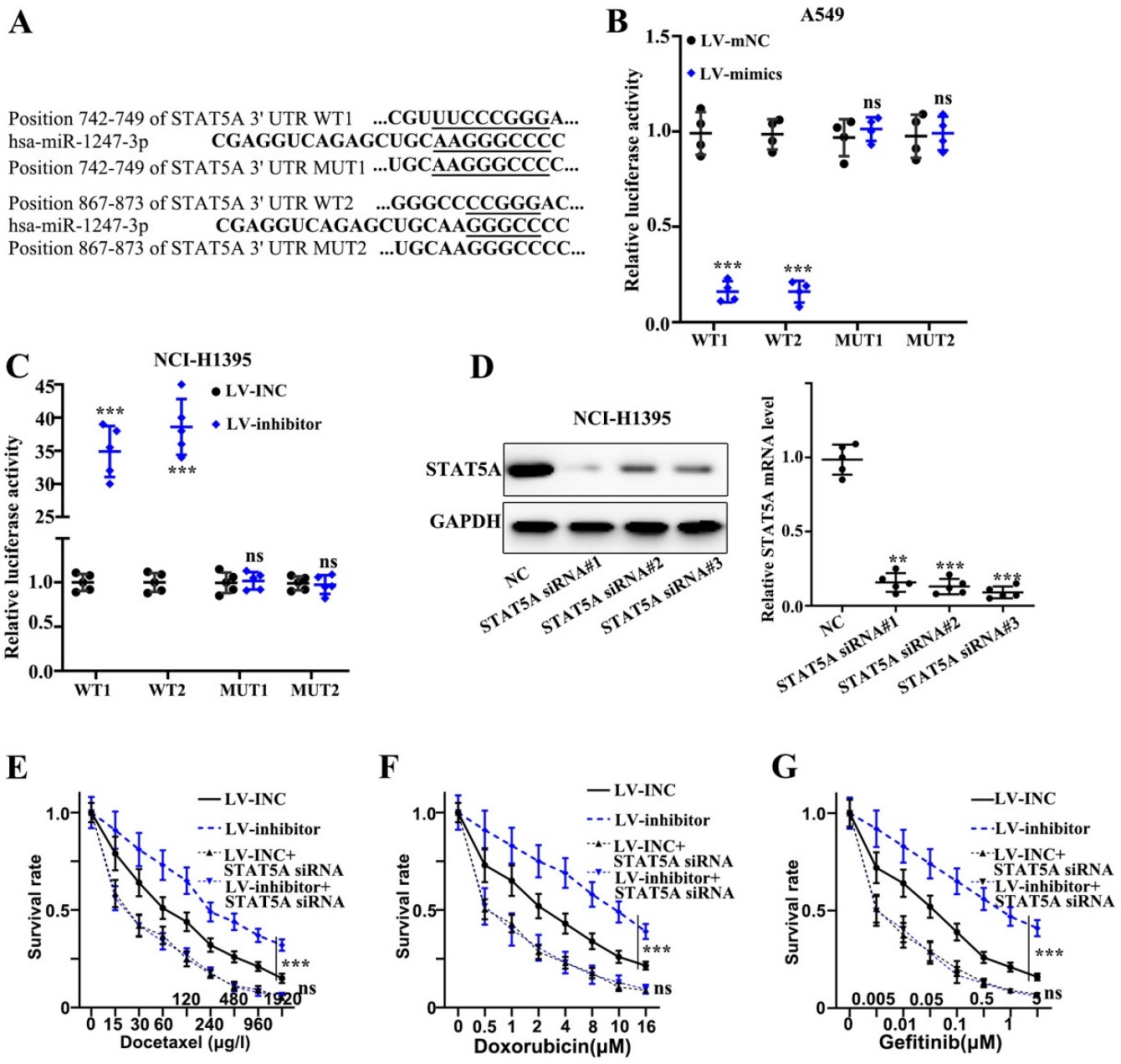
** miR-1247-3p directly binds to STAT5A mRNA 3' UTR. A.** Plasmids, both mutant type and wild-type, of STAT5A mRNA 3' UTR were constructed on the active binding sites of miR-1247-3p and mRNA of STAT5A gene. **B-C.** The binding of miR-1247-3p with wild type plasmid and not the mutant type was verified by the dual luciferase assay. **D.** Sequence of siRNA of STAT5A exhibiting the best interference effect was identified and separated for further experiments. **E.** Higher survival rate of inhibitor group as compared to LV-INC group upon treatment with Docetaxel. No statistical significance between LV-INC+STAT5A siRNA and LV-inhibitor+STAT5A siRNA group was observed. **F.** Higher survival rate of inhibitor group as compared to the LV-INC group upon treatment with Doxorubicin. No statistical difference between LV-INC+STAT5A siRNA and LV-inhibitor+STAT5A siRNA groups was observed. **G.** Higher survival rate of inhibitor group as compared to the LV-INC group upon treatment with Gefitinib. No statistical difference between LV-INC+STAT5A siRNA and LV-inhibitor+STAT5A siRNA groups was observed. ***p<0.001 compared with group NC, LV-INC, or LV-mNC. Lentiviruses-inhibitor normal control (LV-INC), Lentiviruses-inhibitor (LV-inhibitor), Lentiviruses-mimics normal control (LV-mNC), Lentiviruses- mimics (LV-mimics). The experiments were repeated at least 3 independent times.

We found that plasmids, both mutant type and wild-type, of STAT5A mRNA 3' UTR were constructed on the active binding sites of miR-1247-3p and mRNA of STAT5A gene (Figure 5A). Meanwhile, the binding of miR-1247-3p with wild type plasmid and not the mutant type was verified by the dual luciferase assay (p<0.001) (Figure 5B-C). Sequence of siRNA STAT5A exhibiting the best interference effect was identified and separated for further experiments (Figure 5D). As far the stable transfected cell lines are concerned, their chemotherapy resistance was detected after co-transfecting with STAT5A siRNA. A higher survival rate of inhibitor group compared to LV-INC group upon treatment with Docetaxel (p<0.001) was observed (Figure 5E). No statistical difference between LV-INC+STAT5A siRNA and LV-inhibitor+STAT5A siRNA group was observed. A higher survival rate of inhibitor group as compared to the LV-INC group upon treatment with Doxorubicin (p<0.001) was found (Figure 5F). No statistical difference between LV-INC+STAT5A siRNA and LV-inhibitor+STAT5A siRNA groups was observed. A higher survival rate of inhibitor group as compared to the LV-INC group upon treatment with Gefitinib (p<0.001) (Figure 5G). No statistical difference between LV-INC+STAT5A siRNA and LV-inhibitor+STAT5A siRNA groups was observed.

### Down-regulated STAT5A significantly promoted tumor growth

**Figure 6:**
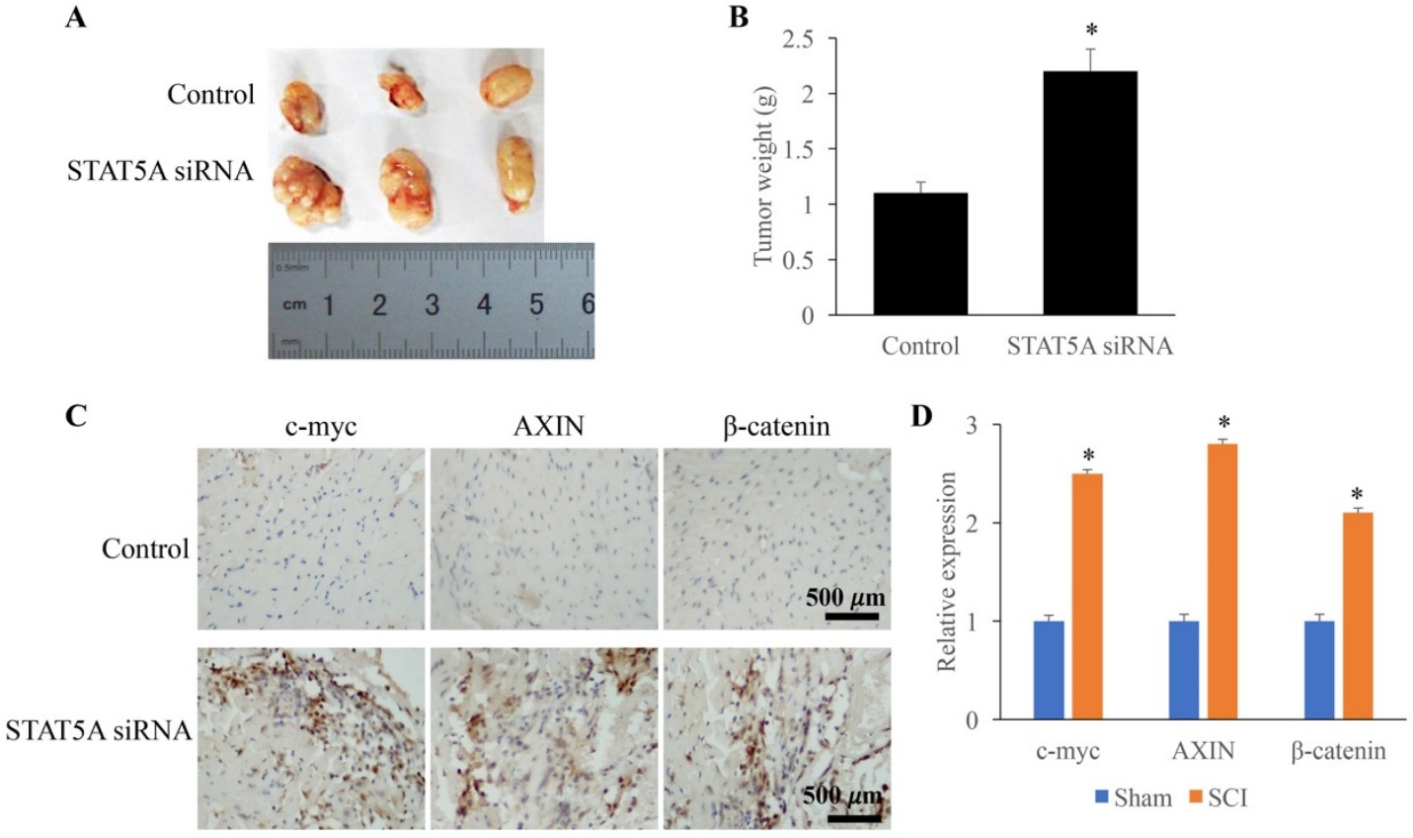
** Down-regulated STAT5A significantly promoted tumor growth. A:** Transplanted tumor model was established to investigate the role of STAT5A on tumor growth. **B:** Down-regulation of STAT5A significantly increased the tumor size. **C:** The levels of c-myc, AXIN, and β-catenin were measured using IHC. **D:** The expression of c-myc, AXIN, and β-catenin were remarkably increased by STAT5A siRNA. *<0.05 Compared with group control. *p<0.05 compared with group control or sham. The animal experiment was performed for one time, and the IHC staining was repeated 3 independent times.

The influence of STAT5A on tumor growth was measured through establishing transplanted tumor model. We found that down-regulation of STAT5A could significantly increase the tumor size (Figure 6A) and tumor weight (Figure 6B). In addition, some molecules related the tumor growth were also investigated. The expression of c-myc, AXIN, and β-catenin were remarkably increased by STAT5A siRNA (Figure 6C-D).

## Discussion

We examined miR-1247-3p expression in lung adenocarcinoma and found it to be reduced in cancerous tissues as well as cell lines. In advanced stages as well as in patients with metastasis, the expression of miR-1247-3p is lower. It can prohibit lung adenocarcinoma cell proliferation and it also can hinder chemotherapy resistance of lung adenocarcinoma cells. Following research showed that it inhibited chemotherapy resistance and it targeted STAT5A, rescue experiments further validated the effects.

There are seven protein family members of signal transducers and transcription activators (STAT), which act as latent cytoplasmic transcription factors. In many human cancers, STAT signaling activation was observed. The STAT family's role in cell differentiation, proliferation, inflammation, and apoptosis has been studied. STAT5 becomes phosphorylated following stimulation by many cytokines and forms a dimer. By direct binding to mtDNA, STAT5 can regulate mitochondrial gene expression 25. STAT5A is a transcription factor in solid cancers involved in cell cycle growth, invasion, and migration 26. It stimulates tumor proliferation, differentiation, and pathogenesis. In previous studies, it was found that, when STAT5A expression was smothered by microRNA, downstream NF-κb signaling and MAPK signaling were found to be significantly increased. AKT phosphorylation was negatively regulated by STAT5A 27. STAT5A is important to regulate stem cells for the production of luminal progenitors that differentiate into mammary alveolar cells. STAT5A has regulated the role of hormones, growth factors, and cytokines in gene expression. In breast or cervical cancer, excessive STAT5A activation triggered by hyper-phosphorylation or self-mutation is a key mechanism for malignant tumor cell proliferation 28. N-alpha-acetyltransferase protein inhibition of STAT5A has led to cell motility suppression and invasion of breast cancer cells 29, 30. Studies have also found that STAT5A has been involved in tumor progression through the intermediation transition of epithelial-mesenchymal. Other studies have found that high STAT5A expression was normally linked with poor ovarian cancer prognosis 31. By stabilizing DGCR8, STAT5 can induce LINC01198 to promote glioma cell proliferation and motility of glioma cells 32. STAT5A deletion in an osteoporosis model related to murine age has been shown to lead to substantially reduced loss of bone. Researches indicate that through balancing adipogenesis and osteogenesis, STAT5A is an important regulator in sustaining bone homeostasis 33.

In previous studies, VEGF signaling pathways, p53, reactive oxygen species, Kras, Notch, inflammatory response, interferon-gamma response, apoptosis, metabolism, complement, and B-cell receptor, chemokine, IL-6/JAK/STAT3, and IL-2/Stat5 were enriched in the high-expression of STAT5A phenotype 34. STAT5A has also been identified as a significant factor viability and growth of prostate cancer cells and plays its role in the metastatic spread of prostate cancer 35, 36. In esophageal cancer, STAT5 is known to control NOX5-S transcriptionally. The results suggest that STAT5A controls the expression of NOX5-L. Studies have found that by influencing the FABP5 expression, which further affects the tumorigenesis of gastric cancer cells, STAT5A can contribute to the process of cellular lipid metabolism. The STAT5A level and the abundance of CD8+ T cells, CD4+ T cells, macrophages, neutrophils, and dendritic cells have been closely inter-related. Activation of STAT5, main component of STAT pathway, is also involved in gastric cancer 37. However, there are some limitations for this study. Only one hospital and 162 patients were selected in this study, and more hospitals and patients are needed for the validation of these findings. In addition, the influence of miR-1247-3p on tumor growth was not investigated *in vivo*.

## Conclusions

We observed a low expression of miR-1247-3p in LUAD. MiR-1247-3p can regulate cell migration and chemotherapy resistance in LUAD cells through targeting STAT5A. Our research led to the novel understanding of LUAD cancer cell migration and chemotherapy resistance. There is potential to exploit miR-1247-3p as novel target for treating lung adenocarcinoma in the future. Meanwhile, this study might provide a novel insight for the prevention and treatment of LUAD through targeting miR-1247-3p/STAT5A axis.

## Figures and Tables

**Table 1:** Clinical characteristics of patients

Features	Number of patients
**Age**	
≤65	70
>65	92
**Sex**	
Male	96
Female	66
**Lymph node status**	
N0	15
N1	75
N2	72
**Tumor metastasis**	
M0	140
M1	22

## Abbreviations

LUAD: Lung adenocarcinoma
PBS: Phosphate buffer saline
FBS: Fetal bovine serum
CCK-8: Cell counting kit-8
